# ANOCVA in R: A Software to Compare Clusters between Groups and Its Application to the Study of Autism Spectrum Disorder

**DOI:** 10.3389/fnins.2017.00016

**Published:** 2017-01-24

**Authors:** Maciel C. Vidal, João R. Sato, Joana B. Balardin, Daniel Y. Takahashi, André Fujita

**Affiliations:** ^1^Department of Computer Science, Institute of Mathematics and Statistics, University of São PauloSão Paulo, Brazil; ^2^Center of Mathematics, Computation, and Cognition, Universidade Federal do ABCSanto André, Brazil; ^3^Hospital Israelita Albert EinsteinSão Paulo, Brazil; ^4^Deparment of Psychology and Princeton Neuroscience Institute, Princeton UniversityPrinceton, NJ, USA

**Keywords:** Analysis of Cluster Variability, silhouette statistic, functional brain network, ABIDE, fMRI

## Abstract

Understanding how brain activities cluster can help in the diagnosis of neuropsychological disorders. Thus, it is important to be able to identify alterations in the clustering structure of functional brain networks. Here, we provide an R implementation of Analysis of Cluster Variability (ANOCVA), which statistically tests (1) whether a set of brain regions of interest (ROI) are equally clustered between two or more populations and (2) whether the contribution of each ROI to the differences in clustering is significant. To illustrate the usefulness of our method and software, we apply the R package in a large functional magnetic resonance imaging (fMRI) dataset composed of 896 individuals (529 controls and 285 diagnosed with ASD—autism spectrum disorder) collected by the ABIDE (The Autism Brain Imaging Data Exchange) Consortium. Our analysis show that the clustering structure of controls and ASD subjects are different (*p* < 0.001) and that specific brain regions distributed in the frontotemporal, sensorimotor, visual, cerebellar, and brainstem systems significantly contributed (*p* < 0.05) to this differential clustering. These findings suggest an atypical organization of domain-specific function brain modules in ASD.

## Introduction

The brain activity is organized in clusters/modules that have different roles in our behavior (Tononi et al., 1999). Alterations in the clustering pattern can be associated with neurologic disorders (Grossberg, 2000; Sato et al., 2016). Thus, it is important to systematically discriminate the clustering structures among different populations. This leads to the problem of how to statistically test the equality of clustering structures of two or more populations and how to identify the features that contribute to the differential clustering structure. These statistical problems were recently solved for a large class of clustering algorithms by using the Analysis of Cluster Variability—ANOCVA (Fujita et al., 2014a).

Here, we provide an implementation of ANOCVA in R for a better dissemination of this technique in the scientific community. ANOCVA was designed to test whether the clustering structures of several populations are equal. Briefly, ANOCVA uses the silhouette statistic (Rousseeuw, 1987) as a measure of variability of the clustering structure of each population and then compares the variability among populations using an idea similar to the classical analysis of variance (ANOVA). To calculate the statistical significance value, we use a bootstrap procedure that was previously shown to control the type I error.

We illustrate the step-by-step application of ANOCVA by analyzing a large functional magnetic resonance imaging (fMRI) data acquired under a resting-state protocol (ABIDE—The Autism Brain Imaging Data Exchange Consortium) composed of 529 controls and 285 patients diagnosed with autism. Subjects with Autism Spectrum Disorders (ASD) have significant differences in the resting state functional connectivity when compared to healthy subjects (for review, see Kana et al., 2011), suggesting that ASD is as a neural systems disorder with disruptions in several distributed neurocognitive networks of brain regions (Ecker et al., 2015). However, most studies describe integration (Washington et al., 2014; Sporns and Betzel, 2016) and segregation (Assaf et al., 2013) as separate processes. Instead, in this study we consider both processes simultaneously using the idea of clusters, where structures within are integrated and structures between are segregated.

## Materials and methods

To formalize ANOCVA, we will first describe the silhouette statistic to define “clustering variability” and then we introduce the ANOCVA. Finally, we describe its implementation and application to ABIDE dataset.

### The silhouette statistic

The silhouette statistic is a measure of how well an item (regions of interest—ROI in fMRI data) is clustered given a clustering algorithm. In other words, it can also be interpreted as a measure of clustering variability (Rousseeuw, 1987). Formally, let χ = {*x*_1_, .., *x*_*N*_} be the *N* ROIs of one subject that are clustered into *C* = {*C*_1_, …, *C*_*r*_} clusters by a clustering algorithm. Denote the dissimilarity between ROIs *x* and *y* by *d*(*x, y*). Let |*C*| be the number of ROIs of *C*. Then, define $d\left(x,C\right)=\frac{1}{\left|C\right|}\sum_{y\in C}d\left(x,y\right)$ as the average dissimilarity of *x* to all ROIs of cluster *C*. Denote *D*_*q*_ ∈ *C* as the cluster to which *x*_*q*_ has been assigned by the clustering algorithm. Define *a*_*q*_ = *d*(*x*_*q*_, *D*_*q*_) (the within dissimilarity of *x*_*q*_) and *b*_*q*_ = min_*C*_*p*_≠*D*_*q*__ *d*(*x*_*q*_, *C*_*p*_) (the smallest between dissimilarity of *x*_*q*_), for *q* = 1, …, *N*. Then, we can measure how well each ROI *x*_*q*_ has been clustered by analyzing the silhouette statistic given by

$$s_{q}=\{\begin{array}{c} \frac{b_{q}\;-\;a_{q}}{\max\;\{b_{q},\;a_{q}\}},\;if\;|D_{q}|\;>1,\;\\ 0,\;if\;|D_{q}|=1. \end{array}$$

The silhouette statistic *s*_*q*_ assumes values from −1 to +1 and its interpretation given by Rousseeuw (1987) is as follows. If *s*_*q*_ ≈ 1, it means *a*_*q*_ ≪ *b*_*q*_, i.e., the ROI *x*_*q*_ has been assigned to an appropriate cluster because the second-best choice cluster is not as close as the actual cluster. If *s*_*q*_ ≈ 0, then *a*_*q*_ ≈ *b*_*q*_. In this case, it is not clear whether ROI *x*_*q*_ should have been assigned to the actual cluster or to the second-best choice cluster because it is equally far away from both. If *s*_*q*_ ≈ −1, then *a*_*q*_ ≫ *b*_*q*_. In other words, the ROI *x*_*q*_ should be assigned to the second-best choice cluster because it lies much closer to it than to the actual cluster. In summary, *s*_*q*_ is a measure of how well the clustering algorithm labeled ROI *x*_*q*_.

### ANOCVA

In the present section, we briefly describe the ANOCVA. For further details, refer to Fujita et al. (2014a). Let *T*_1_, *T*_2_, …, *T*_*k*_ be *k* types of populations (e.g., controls and ASD). For the *j* th population, *n*_*j*_ subjects are collected, for *j* = 1, …, *k*. The items (e.g., ROIs) of the *i* th subject taken from the *j* th population are represented by the matrix *X*_*i, j*_ = (*x*_*i, j*, 1_, …, *x*_*i, j, N*_), where each ROI *x*_*i, j, q*_ (*q* = 1, .., *N*) is a vector containing a time series (the blood-oxygen-level dependent signal).

First, define the (*N* × *N*) matrix of dissimilarities among ROIs of each matrix *X*_*i,j*_ by $A_{i,j}=\left\{d\left(x_{i,j,q},x_{i,j,q^{\prime }}\right)\right\}$, for *i* = 1, …, *n*_*j*_, *j* = 1, …, *k*. Second, let $n=\sum_{j=1}^{k}n_{j}$, then define the following average matrices of dissimilarities:

$$\begin{array}{l} {\bar{A}}_{j}=\frac{1}{n_{j}}\sum_{i\;=\;1}^{n_{j}}A_{i,j}=\frac{1}{n_{j}}\sum_{i\;=\;1}^{n_{j}}\{d(x_{i,j,q},x_{i,j,q\prime })\}\text{ and}\\ \overset{=}{A}=\frac{1}{n}\sum_{j\;=\;1}^{k}n_{j}{\bar{A}}_{j},\text{ where }q,\;q\prime =1,\ldots ,N. \end{array}$$

Next, apply a clustering algorithm on the matrix of dissimilarities $\overset{=}{A}$, to determine the clustering labels $\underset{A}{l=.}$ Finally, compute the following silhouette statistics: $s_{q}^{\left(\bar{\bar{A}},l_{\bar{\bar{A}}}\right)}$ (the silhouette statistic of the *q*th ROI based on the dissimilarity matrix $\overset{=}{A}$ and the labeling $\underset{A}{l=}$) and $s_{q}^{\left({\bar{A}}_{j},l_{\bar{\bar{A}}}\right)}$ (the silhouette statistic of the *q*th ROI based on the dissimilarity matrix ${\bar{\text{A}}}_{j}$ and the labeling $\underset{A}{l=}$), for *q* = 1, …, *N*. The statistical test consists in verifying whether all *k* populations are equally clustered (present the same clustering structure) or if at least one is clustered in a different manner. If the ROIs from all populations *T*_1_, …, *T*_*k*_ are equally clustered, then the quantities $s_{q}^{\left(\bar{\bar{A}},l_{\bar{\bar{A}}}\right)}$ and $s_{q}^{\left({\bar{A}}_{j},l_{\bar{\bar{A}}}\right)}$ must be close for all *j* = 1, …, *k* and *q* = 1, …, *N*.

Given a clustering algorithm and a distance metric, define the following vectors:

$$S=(s_{1}^{\left(\overset{=}{A},l\underset{A}{=}\right)},\ldots ,\;s_{N}^{\left(\overset{=}{A},l\underset{A}{=}\right)}{)}^{\text{T}}\text{ and }S_{j}=(s_{1}^{\left(\bar{A},l\underset{A}{=}\right)},\ldots ,\;s_{N}^{\left(\bar{A},l\underset{A}{=}\right)}{)}^{\text{T}}.$$

Define δ*S*_*j*_ = *S* − *S*_*j*_. We will use the statistic $\Delta S=\sum_{j=1}^{k}\delta {\text{S}}_{j}^{\text{T}}\delta {\text{S}}_{j}$ to build the test statistic. Notice that under the null hypothesis, all *N* ROIs are equally clustered along the *k* populations, i.e., $s_{q}^{\left(\bar{\bar{A}},l_{\bar{\bar{A}}}\right)}$ ≈ $s_{q^{\prime }}^{\left(\bar{A},l_{\bar{\bar{A}}}\right)}$ for all *q* = 1, …, *N* and thus, we expect small Δ*S*. On the other hand, large Δ*S* suggests a rejection of the null hypothesis.

To test the contribution of each ROI for the differential clustering, define $\delta s_{q}\;=s_{q}^{\left(\bar{\bar{A}},l_{\bar{\bar{A}}}\right)}-\frac{1}{k}\sum_{j\;=\;1}^{k}s_{q}^{\left(\bar{A},l_{\bar{\bar{A}}}\right)},$ for *q* = 1, …, *N*. This test consists in verifying whether the *q*th ROI (*q* = 1, …, *N*) is equally clustered among populations. We will use the statistic $\Delta s_{q}=\delta s_{q}^{2}$, for *q* = 1, …, *N* to build the test statistic. Under the null hypothesis, we expect small Δ*s*_*q*_. On the other hand, large Δ*s*_*q*_ suggests a rejection of the null hypothesis.

To compute distributions of Δ*S* and Δ*s*_*q*_ under the null hypothesis, Fujita et al. (2014a) proposed a bootstrap procedure described as follows:

1. Resample with replacement *n*_*j*_ subjects from the entire dataset {*T*_1_, *T*_2_, …, *T*_*k*_} in order to construct bootstrap samples $T_{j}^{*}$, for *j* = 1, …, *k*.
2. Calculate ${\bar{A}}_{j}^{*},\overset{=*}{A},\;s_{q}^{\left(\bar{A},l_{\bar{\bar{A}}}\right)}{}^{*}\text{and }s_{q}^{\left(\bar{A},l_{\bar{\bar{A}}}\right)}{}^{*},\text{for }q\;=\;1,\ldots ,N,$, using the bootstrap samples $T_{j}^{*}$.
3. Calculate ${\hat{\Delta S}}^{*}$ and ${\hat{\Delta s_{q}}}^{*}$.
4. Repeat steps 1 to 3 until the desired number of bootstrap replications is obtained.
5. The *p*-values from the bootstrap tests based on the observed statistics Δ*S* and Δ*S*_*q*_ are the fraction of replicates of ${\hat{\Delta S}}^{*}$ and ${\hat{\Delta s_{q}}}^{*}$ on the bootstrap dataset $T_{j}^{*}$, respectively, that are at least as large as the observed statistics on the original dataset.

### R implementation

ANOCVA is implemented in R and is freely available at the R project website^1^ (package “anocva”).

This implementation requires as input, the functional brain networks (ROIs dissimilarity matrices), a vector of labels describing which individual belongs to which group, the number of clusters, and the number of bootstrap samples.

ANOCVA uses the spectral clustering algorithm to cluster the ROIs (Ng et al., 2002). Internal to the spectral clustering algorithm, we use the *k* -medoids procedure instead of the usual *k* -means because the former is more robust to outliers than the latter (Aggarwal and Reddy, 2013). If the number of clusters is not known a priori, the ANOCVA R package provides the option to estimate it by using the silhouette or the slope statistic (Fujita et al., 2014b). The slope criterion is the difference of the silhouette statistic as a function of the number of clusters. The difference between the slope and silhouette is the fact that by maximizing the silhouette statistic as described by Rousseeuw (1987) the number of clusters is estimated correctly only when the within-cluster variances are equal. The slope criterion is more robust than the silhouette when the within-cluster variances are unequal.

**Figure 1:**
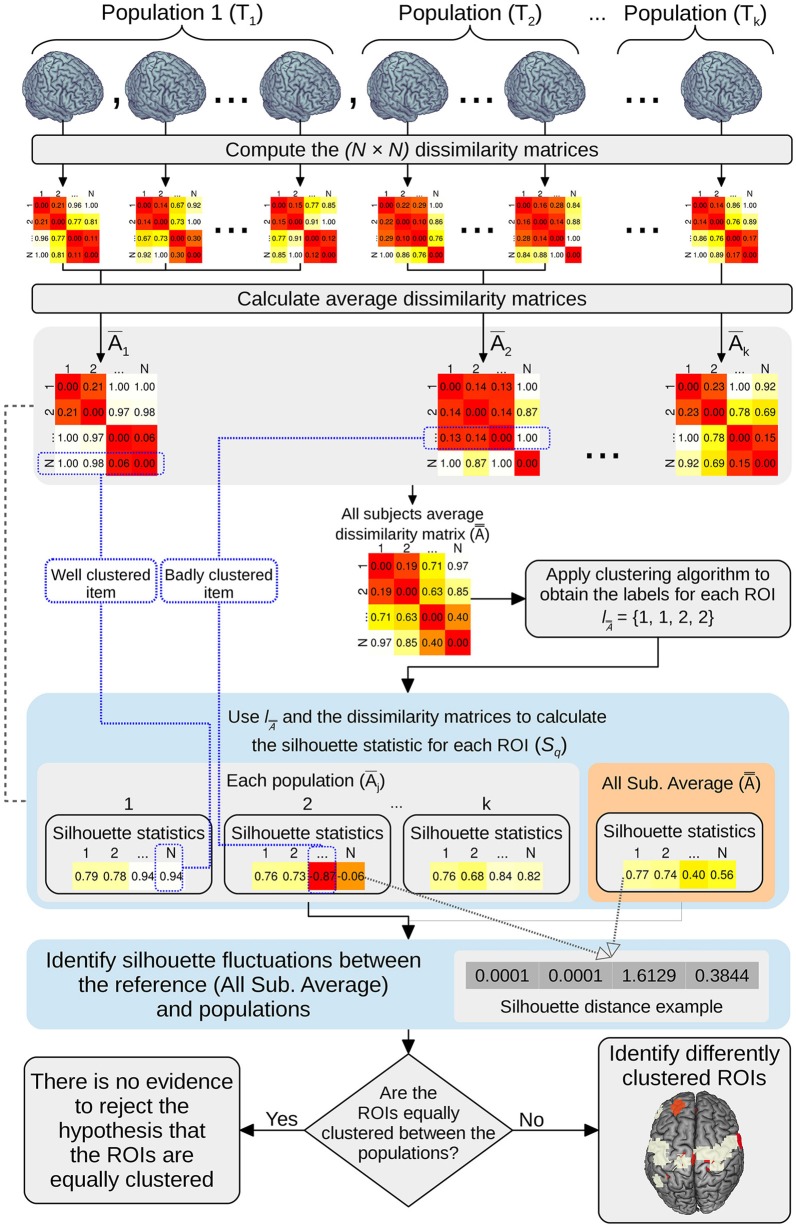
**Pipeline schema of the ANOCVA analysis**.

The output consists in one *p*-value, which represents whether there is at least one group that clusters in a different manner and a vector of *p*-values representing which ROI is differentially clustered among groups. The entire ANOCVA analysis pipeline can be visualized in Figure 1.

### ABIDE data description and pre-processing

The ABIDE Consortium dataset is a large resting state fMRI dataset that includes controls and ASD subjects. It can be downloaded from the ABIDE website^2^. This data was collected in 17 sites that compose the ABIDE Consortium. Data collection was conducted with local internal review board approval, and also in accordance with local internal review board protocols. For further details regarding this dataset, refer to the ABIDE Consortium website.

Data pre-processing and network construction (dissimilarity matrices) were carried out as our previous works (Sato et al., 2015, 2016) using the ABIDE dataset. The final dataset used here is composed of 529 controls (430 males, mean age ± standard deviation of 17.47 ± 7.81 years) and 285 autistic patients (255 males, 17.53 ± 7.13 years).

## Results

The problem that we want to solve is the following. Given *k* populations *T*_1_, *T*_2_, …, *T*_*k*_ where each population *T*_*j*_ (*j* = 1, …, *k*) is composed of *n*_*j*_ subjects, and each subject has *N* items that are clustered, we would like to verify whether the clustering structures of the brain networks of the *k* populations are equal and, if not, which ROIs are differently clustered. In our case, we have *k* = 2 populations with *T*_1_ and *T*_2_ as controls and ASD, respectively. The number of subjects in each population is *n*_1_ = 529 and *n*_2_ = 285, for *T*_1_ and *T*_2_, respectively. The number of ROIs (items) to be clustered is *N* = 316. Since head movement during magnetic resonance scanning may affect statistical analysis, ANOCVA was applied to both “scrubbed” and “not scrubbed” data (Power et al., 2012) with the number of bootstrap samples set to 1000.

**Figure 2:**
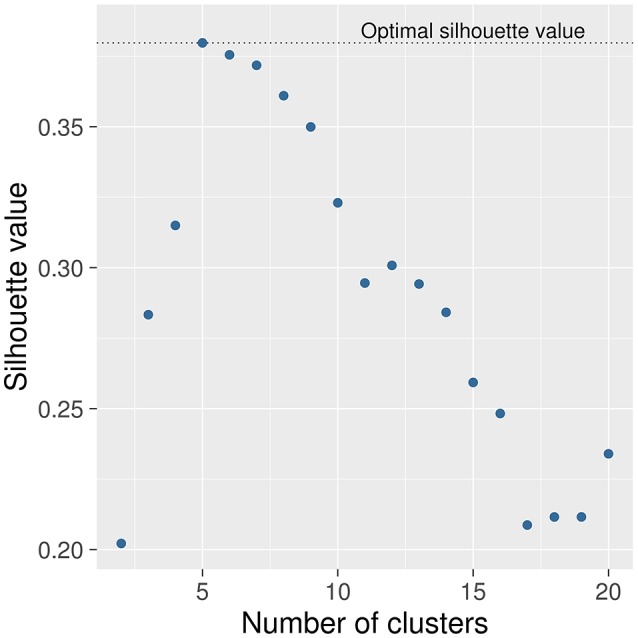
**Selection of the number of clusters**. The number of clusters was selected by using the silhouette criterion. The number of clusters that presented the highest silhouette statistic is five. In other words, the silhouette criterion suggests that this dataset can be split into five sub-networks.

**Figure 3:**
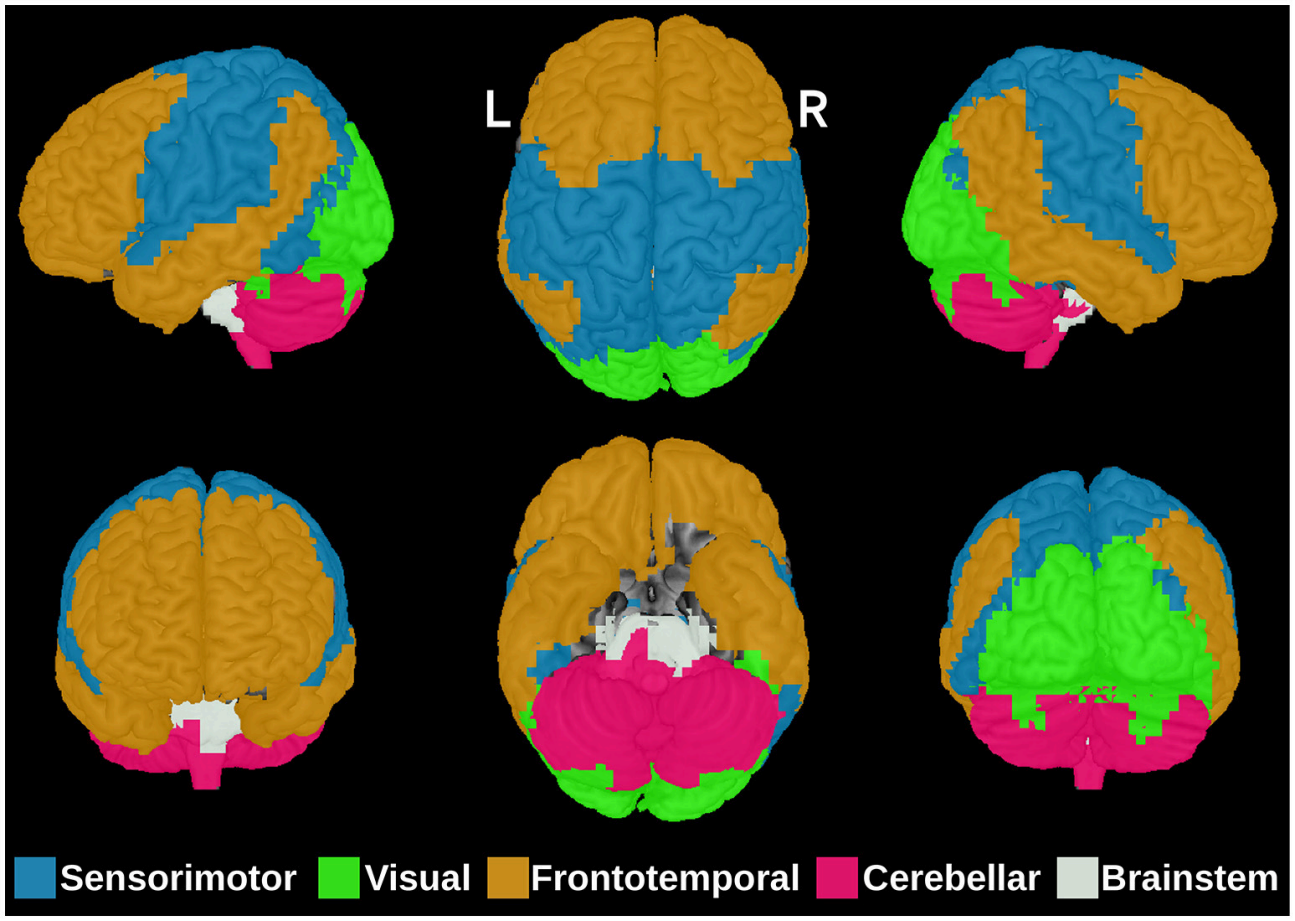
**The five brain sub-networks obtained by the spectral clustering algorithm on the dissimilarity matrix $\overset{=}{A}$**. Each color represents one functional sub-network: sensorimotor (blue), visual (green), frontotemporal (orange), cerebellar (pink), and brainstem (white). R, right; L, Left.

The first step in ANOCVA analysis is the construction of the average dissimilarity matrix $\overset{=}{A}$ and its clustering. The estimated number of clusters by the silhouette criterion was five as depicted in Figure 2. Notice that the highest silhouette statistic was obtained when the number of clusters is five. The sub-networks obtained by applying the spectral clustering on the dissimilarity matrix $\overset{=}{A}$ can be visualized in Figure 3 where each color represents one sub-network (cluster).

**Figure 4:**
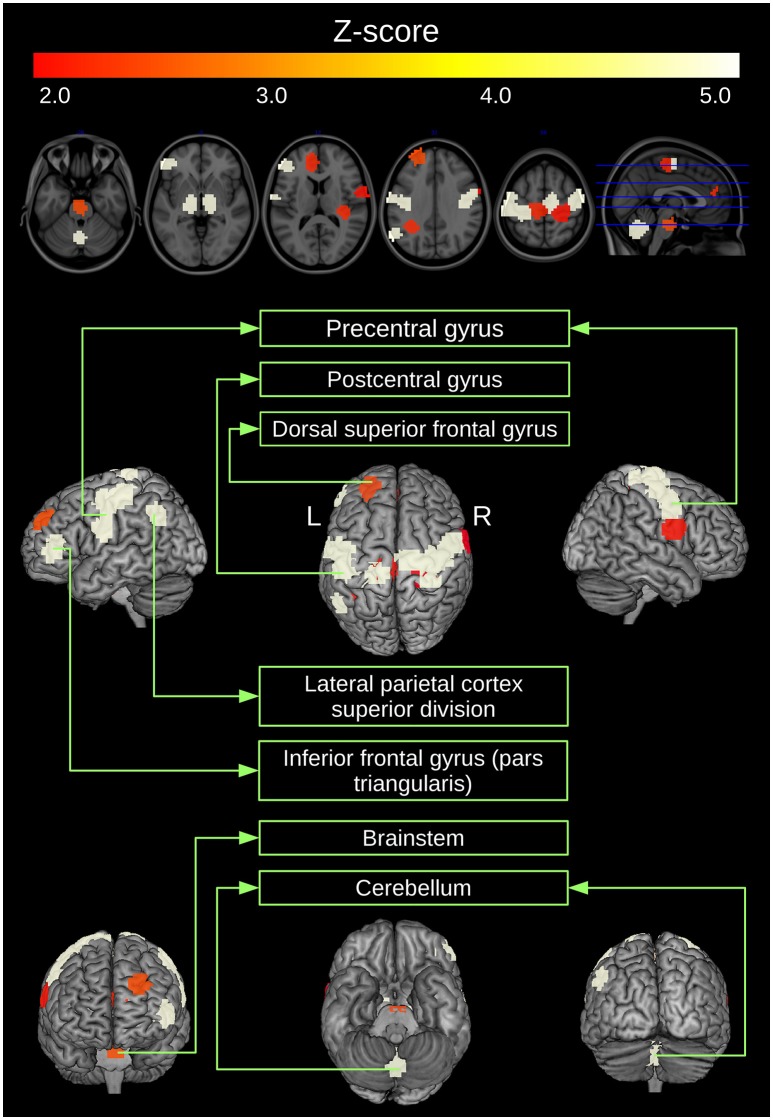
**ROIs clustered in a different manner between controls and ASD**. ROIs that present a *p*-value (obtained by ANOCVA) lower than 5% after Bonferroni correction were converted to z-scores and highlighted.

Then, ANOCVA calculates the silhouette statistic for each ROI by using the labels obtained by clustering the dissimilarity matrix $\overset{=}{A}$ and performs the test. We verified that in fact the entire clustering structure of subjects diagnosed with ASD differs from controls (*p* < 0.001). Next, we tested each ROI to identify which ones significantly contribute to the differential clustering between controls and subjects diagnosed with ASD. ROIs that presented a difference in *p* > 5% between “scrubbed” and “not scrubbed” datasets were excluded for subsequent analysis. Remaining *p*-values were corrected for multiple comparisons by the Bonferroni method. Figure 4 illustrates the statistically significant ROIs at a *p*-value threshold of 0.05 after Bonferroni correction. The highlighted regions include portions of the cerebellum and middle frontal gyrus, pre- and post-central gyri, inferior temporal gyrus, and lateral occipital cortex.

## Discussion

In the current study, we combined spectral clustering analysis with ANOCVA implemented in R to investigate which brain regions are clustered in a different way between controls and ASD groups. Our results suggest that several regions distributed across different neurocognitive systems significantly contributed to the different clustering network structure observed in ASD. First we demonstrated that the spectral clustering method yielded partitions that were well-characterized as functional modules of the brain that have been consistently identified in previous studies using different approaches (Damoiseaux et al., 2006; Power et al., 2011), including the fronto-temporal, sensorimotor, visual, and cerebellar systems. This is consistent with the hypothesis that the spectral clustering algorithm groups anatomically contiguous and also spatially distributed areas with common brain functionalities in the same cluster. Then, using ANOCVA we showed that the superior division of the lateral parietal cortex, precentral, and postcentral gyri, anterior dorsal middle frontal gyrus, and a medial portion of the cerebellum and of the brainstem have a distinct cluster organization between ASD and controls. All these brain regions have been previously identified as presenting ASD-related differences in studies using functional MRI. For example, the recruitment of portions of the precentral and postcentral gyri as well as the cerebellum across sensorimotor tasks are atypical in ASD, and may underlie deficits in fine motor sequencing and visual motor learning observed in autistic individuals (Müller et al., 2001; Mostofsky et al., 2009). Interestingly, these regions have also been implicated in cognitive process crucial for interpersonal interactions such as theory-of-mind (Martineau et al., 2010; Wang et al., 2014). This suggests that these areas are involved in the social communication deficits that are a core clinical feature of ASD. Moreover, the lateral parietal cortex is an important node of the default-mode network, and abnormalities in the connectivity between nodes of this network have been widely investigated in ASD (Kennedy and Courchesne, 2008; Assaf et al., 2010; Weng et al., 2010) giving its associations with social cognition (Buckner et al., 2008). The identification of these regions by our study therefore confirms that they are key brain structures in ASD that may have a role in the development of sub-networks organization in this population.

Head motion is one of the most challenging obstacles in functional connectivity studies involving clinical populations, which usually present high levels of movement. Our attempt to handle this problem was to apply the scrubbing method proposed by Power et al. (2012), which discards scans acquired under excessive head motion. However, although this approach may reduce the influence of movement artifacts, they may still be present in the scrubbed data. Thus, we opted for a more conservative approach, which consisted in excluding the regions where the *p*-values were more sensitive to scrubbing. We assumed that the analyses of these regions were more vulnerable to artifacts and thus they were removed. This approach is also helpful to reduce the number of multiple comparisons, by excluding the less reliable tests. Another important limitation to be mentioned is that the ABIDE data is multicentric with heterogeneous acquisition parameters across sites. We minimized the site effect by removing it in the pre-processing stage of the data. Finally, all analyses are based on the CC400 atlas (Craddock et al., 2012), obtained by using a functional parcellation. Since other atlases are different on ROIs size, number of ROIs and spatial location, the parcellation choice is expected to influence our findings. However, this variability does not invalidate the results obtained with CC400 because the procedures adopted here are conservative (regarding type I error control). Finally, an important future question for the presented results is whether the contribution of these specific brain regions to a differential network clustering in ASD is static or may exhibit dynamic changes during rest (Hutchison et al., 2013).

## Author contributions

MV, JS, DT, and AF designed the work. MV pre-processed and analyzed the data. JS and JB interpreted the results. All authors drafted the work, read, and approved the final version of the manuscript.

## Funding

MV was supported by CAPES and CNPq Fellowships. JS was supported by State of São Paulo Research Foundation—FAPESP (#2013/10498-6). DT was partially supported by Pew Latin American Fellowship and Ciência sem Fronteiras Fellowship (CNPq #246778/2012-1). AF was partially supported by FAPESP (#2014/09576-5, #2013/01715-3, #2015/01587-0, #2016/13422-9, and #2013/03447-6), CNPq (#304020/2013-3 and #473063/2013-1), and NAP eScience—PRP—USP.

### Conflict of interest statement

The authors declare that the research was conducted in the absence of any commercial or financial relationships that could be construed as a potential conflict of interest.

## Footnotes

^1^www.r-project.org

^2^http://fcon_1000.projects.nitrc.org/indi/abide/
